# Neural Architecture for Feature Binding in Visual Working Memory

**DOI:** 10.1523/JNEUROSCI.3493-16.2017

**Published:** 2017-04-05

**Authors:** Sebastian Schneegans, Paul M. Bays

**Affiliations:** Department of Psychology, University of Cambridge, Cambridge CB2 3EB, United Kingdom

**Keywords:** cued recall, feature binding, population coding, visual working memory

## Abstract

Binding refers to the operation that groups different features together into objects. We propose a neural architecture for feature binding in visual working memory that employs populations of neurons with conjunction responses. We tested this model using cued recall tasks, in which subjects had to memorize object arrays composed of simple visual features (color, orientation, and location). After a brief delay, one feature of one item was given as a cue, and the observer had to report, on a continuous scale, one or two other features of the cued item. Binding failure in this task is associated with swap errors, in which observers report an item other than the one indicated by the cue. We observed that the probability of swapping two items strongly correlated with the items' similarity in the cue feature dimension, and found a strong correlation between swap errors occurring in spatial and nonspatial report. The neural model explains both swap errors and response variability as results of decoding noisy neural activity, and can account for the behavioral results in quantitative detail. We then used the model to compare alternative mechanisms for binding nonspatial features. We found the behavioral results fully consistent with a model in which nonspatial features are bound exclusively via their shared location, with no indication of direct binding between color and orientation. These results provide evidence for a special role of location in feature binding, and the model explains how this special role could be realized in the neural system.

**SIGNIFICANCE STATEMENT** The problem of feature binding is of central importance in understanding the mechanisms of working memory. How do we remember not only that we saw a red and a round object, but that these features belong together to a single object rather than to different objects in our environment? Here we present evidence for a neural mechanism for feature binding in working memory, based on encoding of visual information by neurons that respond to the conjunction of features. We find clear evidence that nonspatial features are bound via space: we memorize directly where a color or an orientation appeared, but we memorize which color belonged with which orientation only indirectly by virtue of their shared location.

## Introduction

How do we remember which visual features belong together in a briefly glimpsed scene, and how do we keep the features of different objects separate from each other? This problem of feature binding in visual working memory has received significant attention in the psychological and neuroscientific literature (Treisman, 1996), yet there is still no consensus regarding the behavioral signatures of feature binding, nor the underlying neural mechanism.

Two fundamentally different mechanisms have been proposed. In the first, representations of the different features of an object in separate neural populations are bound through synchronization of their spiking activity (von der Malsburg, 1999; Raffone and Wolters, 2001). However, experimental evidence for such a functional role of synchronization in feature binding is limited and controversial (Shadlen and Movshon, 1999; Palanca and DeAngelis, 2005). In the second approach, binding is achieved through conjunctive coding in populations of neurons sensitive to multiple features of an object (Johnson et al., 2008; Matthey et al., 2015; Schneegans et al., 2016). Conjunctive coding is well established at most levels of visual processing in the cortex (Rao et al., 1997; Op De Beeck and Vogels, 2000), with the most prevalent form being combined sensitivity for a nonspatial feature and stimulus location.

So far, both of these accounts have addressed binding in working memory only on a qualitative level without accounting for quantitative behavioral data, while conversely, empirically grounded models of visual working memory in psychology remain agnostic regarding the neural binding mechanism (Kahneman et al., 1992). This discrepancy may stem from the difficulty of distinguishing binding errors from failures to memorize individual features in behavioral studies.

A possible route to overcome this limitation is offered by cued recall tasks (Wilken and Ma, 2004), in which subjects must report on a continuous scale the feature of a cued item from a briefly presented sample array (e.g., item color cued by location). The graded response yields more information than the binary same/different decision in classical change-detection tasks, and allows discriminating between different types of errors. It has been found that a significant proportion of responses with large deviations from the target feature can be attributed to swap errors (Bays et al., 2009), in which subjects report the feature value of an item that is not the cued target. These errors reflect a specific failure in retrieving the correct item from working memory, and can therefore be used to assess the binding between cue and report features.

In the present study, we combine novel experimental results from cued recall tasks with computational modeling to elucidate the mechanism of feature binding in visual working memory. We build on recent findings which demonstrate that decoding from a neural population representation of visual features (Pouget et al., 2000) corrupted by random noise can account for the specific pattern of response errors in working-memory tasks (Bays, 2014). We extend this model by conjunctive coding to capture the binding of multiple features. With this model, we provide an integrated and neurally plausible account of swap errors and response variability in cued recall tasks.

Inspired by previous findings from change-detection tasks indicating a privileged status of location in feature binding (Treisman and Zhang, 2006), we then employed this model to elucidate the concrete role of location in binding other features. In the first form, an object's color and orientation are bound directly through a conjunctive population code. In the alternative form, color and orientation are each bound to an object's location through conjunctive coding, but are bound to each other only via shared locations. We compared predictions of both models to behavioral results in a cued recall task with one spatial and one nonspatial response. We found that the pattern of error correlations was fully consistent with binding via space, but inconsistent with direct binding between color and orientation.

## Materials and Methods

### 

#### 

##### Experiment 1.

Experiment 1 is a cued recall task that tests memory for color-location bindings. Eight participants (three males, five females; aged 20–33 years) participated in the study after giving informed consent, in accordance with the Declaration of Helsinki. All participants reported normal color vision and had normal or corrected-to-normal visual acuity. Stimuli were presented on a 21 inch CRT monitor with a refresh rate of 130 Hz. Participants sat with their head supported by a forehead and chin rest and viewed the monitor at a distance of 60 cm. Eye position was monitored on-line at 1000 Hz using an infrared eye tracker (SR Research).

**Figure 1.**
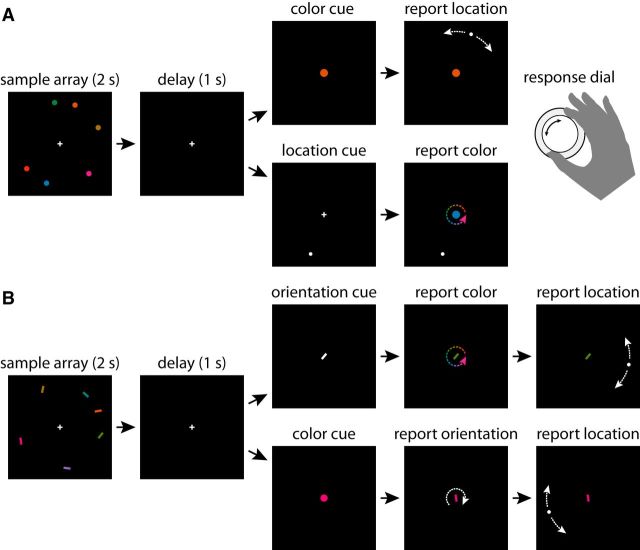
Behavioral tasks. ***A***, Experiment 1: cued recall task with a single response. On each trial, a sample array was presented consisting of six discs with randomly chosen colors and locations on a circle. On report-location trials, participants were shown a color from the sample array and used a dial to move a disc to the matching location. On report-color trials, participants were shown a location from the sample array and used the dial to select the matching color. ***B***, Experiment 2: cued recall task with two responses. The sample array for each trial consisted of six bars with randomly chosen colors, orientations, and locations. On orientation-cue trials, participants were shown an orientation from the sample array and had to sequentially report the matching color and matching location using the response dial. On color-cue trials, participants were shown a color and had to report both matching orientation and location. White/colored arrows indicate possible adjustments of the probe stimulus and are not part of the display.

Each trial began with the presentation of a central white fixation cross (diameter, 0.75° of visual angle) against a black background. Once a stable fixation was recorded within 2° of the cross, a sample array consisting of six colored discs (0.5° radius) was presented for 2 s (Fig. 1*A*). We chose a long presentation time to ensure that recall performance would not be reduced due to incomplete encoding of the sample array (Bays et al., 2009, 2011b). Each colored disc was positioned on an invisible circle, radius 6°, centered on the fixation cross. Locations were chosen at random, with the proviso that every disc was separated from its neighbors by a minimum of 10° on the circle. Colors were selected from a color wheel, defined as a circle in Commission Internationale de l'Eclairage (CIE) L*a*b* coordinates with constant luminance (L* = 50), center at a* = b* = 20, and radius 60. Colors were chosen at random with a minimum separation between discs of 10° on the color wheel.

After the sample array, the display was blanked for 1 s and then a cue display was presented. One of the discs from the sample array, chosen at random, was selected as the target. In the report-location condition (50% of trials), the cue consisted of a centrally presented disc (radius 0.75°) matched in color to the target. Using an input dial (PowerMate USB Multimedia Controller, Griffin Technology), participants adjusted the location of a second disc (white, radius 0.25°) on the invisible circle until it matched the recalled location of the target. In the report-color condition (50% of trials), the cue consisted of a disc (white, radius 0.25°) presented at the location of the target. Participants used the input dial to adjust the color of a centrally presented disc (radius 0.75°), cycling through the color wheel until it matched the recalled color of the target.

Responses were not timed, and subjects were instructed to be as precise as possible. Any trial on which gaze deviated >2° from the central cross before the cue display was aborted and restarted with new feature values. Each subject completed one block of 160 trials for each condition, with the order of blocks counterbalanced across subjects.

##### Experiment 2.

Experiment 2 is a cued recall task in which two features of a cued item are reported, allowing us to test binding between multiple feature dimensions simultaneously and to investigate the role of location in binding nonspatial features. Eight participants (one male, seven females, aged 20–43 years) participated in the experiment after giving informed consent. One additional participant was excluded for persistent failure to maintain fixation. All participants reported normal color vision and had normal or corrected-to-normal visual acuity. Materials and procedures were the same as in Experiment 1, except for the following changes to sample array and report phases: the sample array in Experiment 2 consisted of six colored oriented bars (length 1°, width 0.25°; Fig. 1*B*). Colors and locations were chosen randomly in the same way as in Experiment 1. Orientations were likewise chosen at random, with a minimum separation of 5° between the orientations of different bars (considering the space of unique bar orientations covers only 180°, a 5° separation was chosen to match the 10° used for color and location).

After the presentation of the sample array and a 1 s blank, a cue was presented centrally to indicate which bar from the sample array was the target. In the color-cue condition (50% of trials), the cue was a disc (radius 0.5°) matched in color to the target. Participants had to sequentially indicate the orientation and the location of the target using a single input dial. During the orientation report phase, as soon as the input dial was turned, the central colored disc changed into a bar (length 1°, width 0.25°) of the same color and with random orientation. Participants adjusted the orientation of that bar using the input dial to match the recalled orientation of the target. During the location report phase, a white disc (radius 0.25°) appeared on the invisible circle when the input dial was first turned. Participants adjusted its position on the circle until it matched the recalled location of the target. Participants ended each report phase by depressing the input dial to confirm their response.

In the orientation-cue condition (50% of trials), the cue was a centrally presented white bar (length 1°, width 0.25°) matched in orientation to the target. Participants had to sequentially indicate the color and the location of the target. During the color report phase, the white bar changed to a random color as soon as the input dial was turned. Participants used the input dial to adjust the color of the bar until it matched the recalled color of the target bar. The location report phase proceeded in the same way as in the color-cue condition.

Each subject completed 120 trials in each condition. The order of conditions and the initial order of the two report phases within each condition was counterbalanced across subjects. The order of report phases was then switched after half of the trials in each condition.

##### Analysis.

Stimulus features were analyzed and are reported with respect to the circular parameter space of possible values, [−π, π) radians. Orientation values were scaled up to cover the same range [−π, π) as color-hue values and angular location to allow easier comparison of results across feature dimensions. Recall error for each trial was calculated as the angular deviation between the feature value reported by the participant and the true value. Recall variability was measured by the circular SD as defined by Fisher (1995): σ = $\sqrt{-2\log R}$, with *R* being the length of the mean resultant vector.

The influence of nontarget items was assessed by examining the deviation of responses from nontarget feature values (see Figs. 4*B*,*C*, 5*B*,*C*). Because of the minimum separations between stimuli in feature space, the distribution of deviations expected by chance (i.e., if nontarget values had no impact on response) was not uniform. To obtain the distribution of deviations expected by chance, we used a randomization method: for each subject and condition, deviations of nontarget feature values from target feature values were randomly shuffled, and deviations of responses from the shuffled nontargets were recorded. Averaged over 1000 repetitions, the distribution of response deviations provided an estimate of the chance distribution. This was subtracted from observed response frequencies to produce the corrected-for-chance histograms in Figures 4*B* and 5*B*. Chance values of mean absolute deviation (see Figs. 4*C*, 5*C*, dashed line) were calculated from the randomized deviations for comparison with observed values.

For Experiment 2, we additionally classified trials according to whether the spatial response was directed to the target (a spatial target trial) or one of the nontarget items (a spatial swap trial). To this end, we fit a neural population model only to the spatial responses of each subject. Based on the model fits, we computed for each trial the probability that each item had been selected for spatial response generation, given the actual response location and the locations of all items in the sample array (see Eq. 27). We classified a trial as a spatial target trial if this probability reached 75% for the target item, and as a spatial swap trial if the probability reached 75% for any single nontarget item; trials in which neither condition was fulfilled were classified as ambiguous and not analyzed further. We then determined separately for spatial target and spatial swap trials the distribution of response errors in the nonspatial response (color or orientation). For the spatial swap trials, we additionally determined the distribution of response deviations from the feature value of the spatially selected item (i.e., the nontarget item to which the spatial response was most likely directed).

##### Population-coding model.

For the present work, we built on a previous model of population coding for memorizing individual feature values (Bays, 2014), and adopted key mechanisms of that model. During presentation of a sample array, the memory features of each item are encoded in the activity of a population of neurons: the relationship between an item's feature and each neuron's mean firing rate is determined by the neuron's preferred feature value and its tuning function, which we assume to be normal. For recall, the memorized feature values are read out through maximum likelihood decoding, i.e., the decoder observes the activity of the population and reports whichever feature value makes that particular pattern of activity most likely. Recall errors are explained by random noise in neural activity that causes deviations between encoded and decoded feature values. The model assumes that total neural activity is normalized, i.e., held constant over changes in the amount of information encoded. So, for larger memory arrays, there are fewer spikes encoding each item's feature, leading to poorer recall performance, as observed empirically.

The decoding from neural population activity has been shown to reproduce the quantitative details of error distributions in cued recall tasks (Bays, 2014). These distributions show specific deviations from normality, including an increased proportion of large deviations from the memorized value (long tails in the distribution), accounting for response errors that could be interpreted as random guesses. The proportion of such large errors in the model increases as the number of spikes per item decreases, e.g., due to higher set sizes.

**Figure 2.**
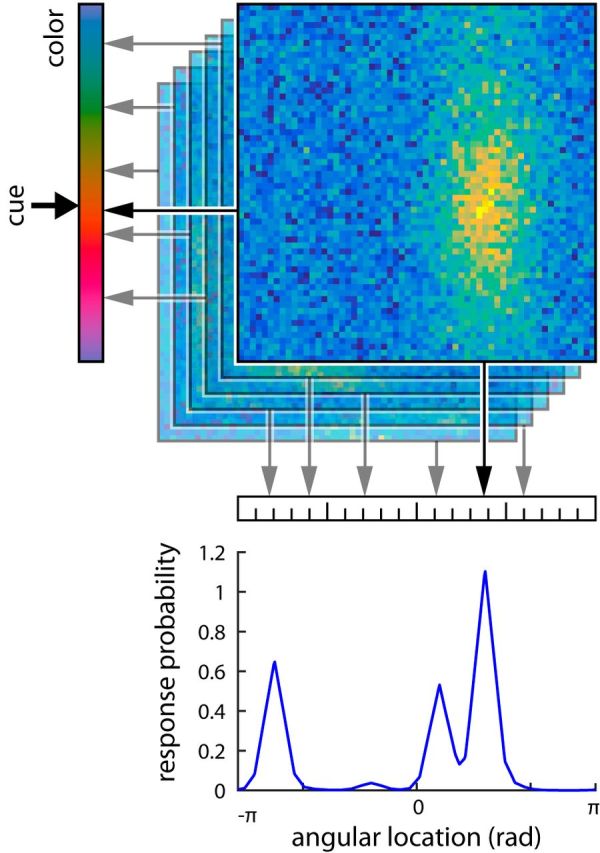
Neural population model. The location and color of each item in the sample array is encoded in the activity of a neural population with conjunctive coding, with added random noise (noise level is reduced in the color-coded activity plots compared with actual simulations to make coding of features more visible). Arrows indicate the estimated feature values for each item obtained by maximum likelihood decoding. For the report-location condition, the memorized item whose decoded color is closest to the cue color (thick arrow) is selected, and its decoded location is produced as a response. The bottom plot shows the distribution of response probabilities derived from the model for the sample array and color cue shown in Figure 1*A*. Note that the lesser peaks reflect swap errors, occurring when a nontarget item is selected because its decoded color value is closest to the cue color.

To address the problem of feature binding and account for swap errors in cued recall tasks, we extend this approach by considering a population code for feature conjunctions (Fig. 2). Each neuron in the population has a preferred value and associated tuning curve for two features (in the basic model, the cue and report features in a cued recall task), and the tuning curves of all neurons cover the two-dimensional space of possible feature conjunctions. For each item to be memorized, a separate population activity is computed based on the item's feature combination, and modulated by random noise. During a cued recall, both the cue and report features of each memorized item are estimated by maximum likelihood decoding from its population representation. The item whose decoded cue feature value is closest to the given cue is selected, and its decoded report feature value is produced as the response.

Compared to previous models, we introduce several simplifications to make the mathematical analysis of the model more feasible. While the original population coding model for individual features was based on spiking neurons with Poisson noise (Bays, 2014), we use rate coding with additive Gaussian noise. We also do not combine the representations of individual items into a single population representation, as was done in a previous “palimpsest” model of feature binding based on conjunctive population coding (Matthey et al., 2015). While arguably more neurally realistic, the palimpsest approach makes it computationally infeasible to perform a maximum likelihood decoding of neural activities for a single trial, and only allows estimation of average error rates based on aggregate effects of nontarget items. For the present model, in contrast, we numerically compute an explicit distribution of response probabilities for each trial. We can thereby fit the model directly to empirical data and make specific predictions about swap errors depending on the properties of target and nontarget items in each trial.

**Figure 3.**
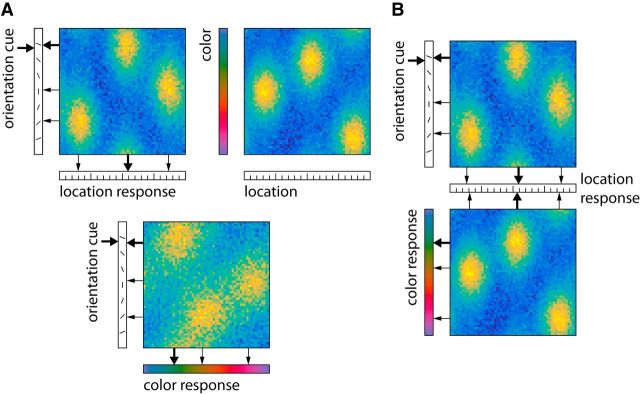
Model architectures for binding multiple nonspatial features. Model depictions are schematized by superimposing population code representations for individual items, and activation patterns are shown for a reduced sample array with only three items. Decoded feature values are shown as arrows, with thicker arrows indicating feature values of selected item. ***A***, Direct-binding model for performing the task in the orientation-cue condition. The population for color–location conjunctions is not used here, but is required for the color-cue condition. ***B***, Spatial-binding model for the same task. This model uses only two conjunctive representations to solve both task conditions.

We further extend the model to the binding of multiple visual features by combining several conjunctive population codes that each bind two feature dimensions. We consider two possible architectures. In the direct-binding model (Fig. 3*A*), one conjunctive population exists for each pair of feature dimensions, explicitly representing binding between the two features. In the spatial-binding model (Fig. 3*B*) a single-feature dimension—namely, spatial location—takes a privileged role in binding all other features together. A conjunctive representation exists binding each nonspatial feature to location, but different nonspatial features are bound to each other only via their shared location. We compare these two architectures based on their performance in fitting behavioral data from the double-report task in Experiment 2.

##### Formal description of the model.

To explain binding between two visual features in working memory, we considered responses of a population of *M* neurons to the presentation of *N* stimuli with cue-dimension features ψ̌ and report-dimension features θ̌. Mean firing rate of the *i*th neuron associated with item *j* is defined as a bivariate function of the item's features according to the following equation (Eq. 1):

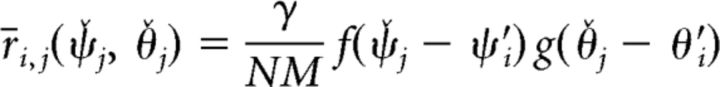
 Neural activity is normalized over the number of neurons *M* and the number of memorized items *N*, and scaled with a gain parameter γ (a measure of the total activity in the population representation). This free parameter globally affects decoding precision and in particular determines the proportion of decoded values with large deviations from the encoded ones. The dependence of neural activity on the encoded feature values is described as a product of tuning functions *f* and *g* with associated preferred values ψ′*_i_* and θ′*_i_* for the two feature dimensions. We considered (non-normalized) von Mises tuning curves of the following form (Eq. 2):

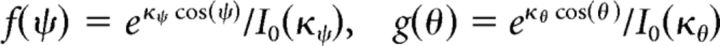
 where κ_ψ_ and κ_θ_ are tuning width parameters that affect decoding precision separately in the two feature dimensions, and *I*_n_(·) is the modified Bessel function of the first kind. The activity of each neuron is corrupted by independent Gaussian noise expressed as follows (Eq. 3):

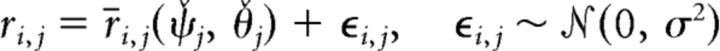
 Setting σ^2^ = γ/(*NM*) approximates Poisson noise (variance is equal to the mean firing rate).

Maximum likelihood decoding from each item's population representation results in feature estimates expressed as follows (Eq. 4):

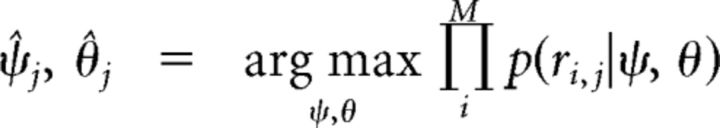
 Recall of target item *t* was tested by presentation of cue feature ψ̌*_t_*. The model returned the decoded report-dimension feature corresponding to the item with decoded cue feature most similar to ψ̌*_t_*, i.e., θ̂*_u_* where

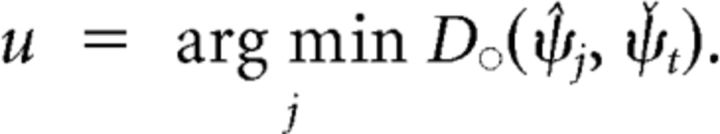
 Here and in the following, *D*_o_ denotes the minimum distance between two angles on a circle, yielding a value in the range [0, π].

##### Estimating decoding probabilities by sampling.

While the equations above provide a complete description of the model, further analysis is needed to obtain predictions of the model and fit them to data. As a first step toward computing response probabilities from the model, we need to estimate the probability distribution *p*(ψ̂*_j_*, θ̂*_j_*) for obtaining values (ψ̂*_j_*, θ̂*_j_*) in the maximum likelihood decoding of the population representation. We do this by sampling, i.e., obtaining many decoded value pairs (ψ̂*_j_*, θ̂*_j_*) from a population code with random noise. In the following, we derive a method to efficiently draw samples from *p*(ψ̂*_j_*, θ̂*_j_*) in a way that does not require explicitly simulating the maximum likelihood decoding of a neural population representation.

From Equation 3, we see that the probability *p*(*r_i,j_*|ψ, θ) in Equation 4 follows a normal distribution with variance σ^2^ around the mean firing rate *r̄_i,j_*(ψ, θ) expressed as follows (Eq. 6):

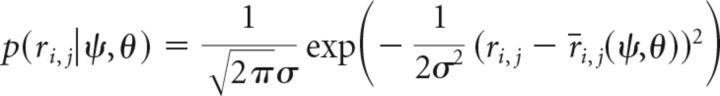
 We insert this into Equation 4, and simplify the equation by omitting constant factors and taking the logarithm of the maximized expression (neither operation affects the resulting arguments of the maximum) expressed as follows (Eq. 7):

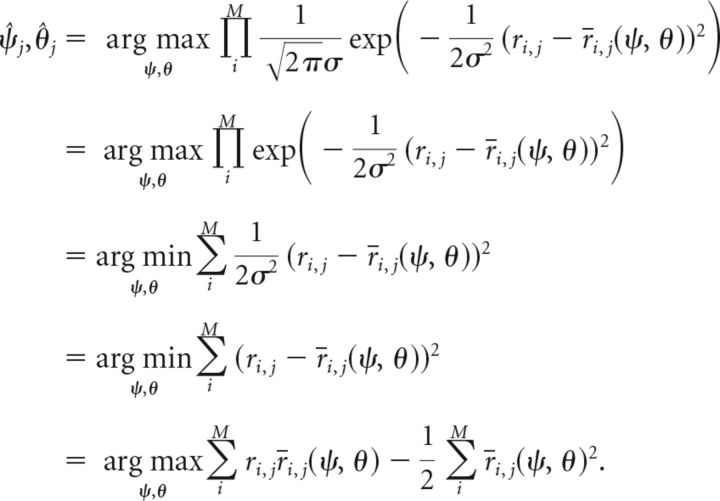
 If we assume dense uniform coverage of the feature space by the neural population (i.e., each point of the feature space is equally and identically covered by neural tuning curves), the second term in the above equation is constant and can be ignored. So we have the following (Eq. 8):

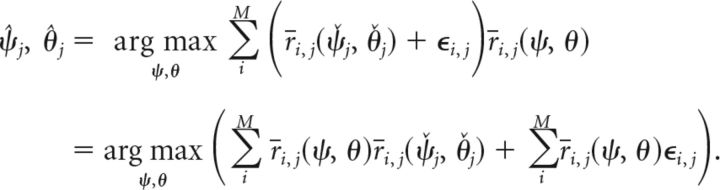
 The expression being maximized here follows a multivariate normal distribution—namely, an infinite-dimensional normal distribution, with each sample drawn from it being itself a distribution over the continuous two-dimensional space of possible cue and report feature values. This can be seen as follows: the second summand in Equation 8 is a sum of normally distributed random variables ε*_i,j_* ∼ 𝒩(0, σ^2^), weighted with the mean neural firing rates for feature values (ψ, θ). Thus, for each point in the two-dimensional feature space, this term is itself a normally distributed random variable with mean zero. The first summand depends only on the mean neural firing rates for a pair of feature values, and on the mean neural firing rates for the actually encoded feature values (ψ̌*_j_*, θ̌*_j_*). The first summand can therefore be considered fixed if these are known, and yields the mean value of the multivariate normal distribution at the point (ψ, θ), expressed as follows (Eq. 9):

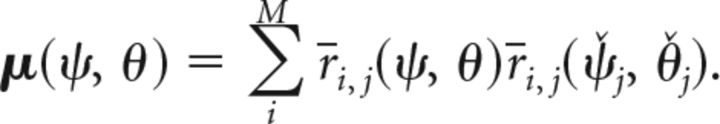
 The covariance between the random variables for two points (ψ_a_, θ_a_) and (ψ_b_, θ_b_) in the two-dimensional feature space depends on the overlap of neural tuning curves at these points. We can compute it from the second summand in Equation 8 and obtain the following (Eq. 10):

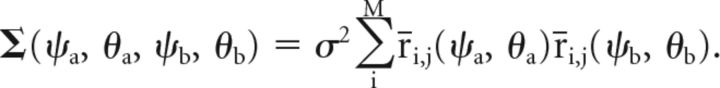
 These equations for mean and covariance fully define the multivariate normal distribution. We can then generate samples of decoded features values (ψ̂*_j_*, θ̂*_j_*) by drawing samples ***Y***(ψ, θ) from this distribution (which are themselves distributions over two-dimensional space) and by determining the arguments of the maximum from these. Rewriting Equation 8 in this fashion yields the following (Eq. 11):

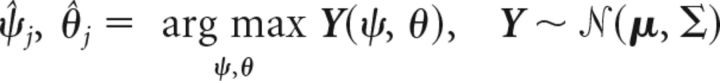
 To sample from the multivariate normal distribution, we need to further resolve the equations for mean and covariance. Inserting the definition of mean firing rates *r̄_i,j_* from Equation 1 yields the following (Eqs. 12 and 13):

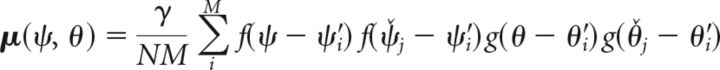


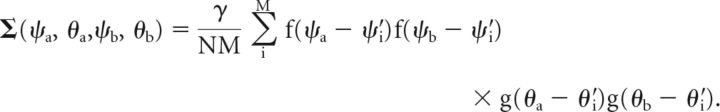
 Again assuming dense uniform coverage of the underlying feature space by neural tuning curves, we can write this in a continuous fashion as follows (Eqs. 14 and 15):

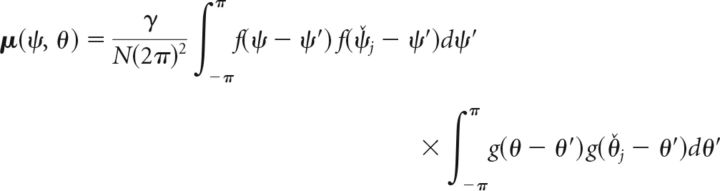


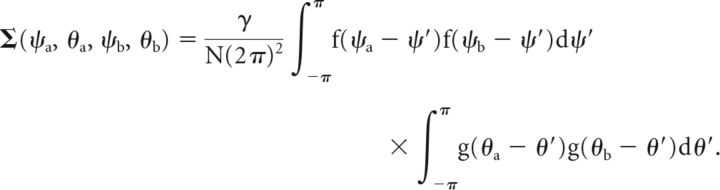
 We can resolve the products of von Mises functions in these expressions using the following general equation (Eq. 16) that holds for any von Mises function h(ω) = *e*^κcos(ω)^/*I*_0_(κ):

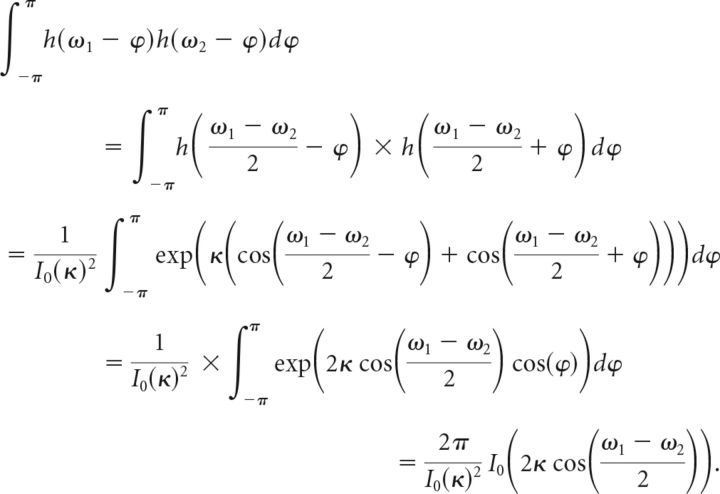
 Based on the definition of the neural tuning functions *f* and *g* in Equation 2, we can use this to rewrite Equations 14 and 15, and obtain as final expressions the following for the mean and covariance of the multivariate normal distribution (Eqs. 17 and 18):

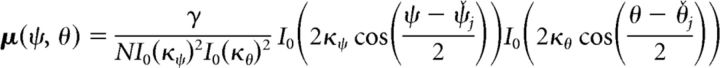


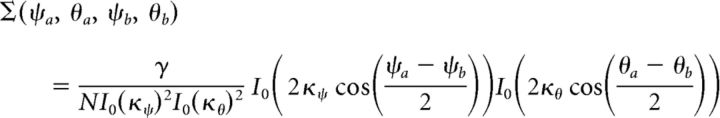
 To generate samples of decoded values, we discretized the space [−π, π) into 36 bins for both the cue and the target feature. We sampled from the multivariate normal distribution ***Y***(ψ, θ) over this discretized space using Cholesky decomposition (cholcov in Matlab). We obtained a two-dimensional histogram of decoded values by generating 10^6^ value pairs (ψ̂*_j_*, θ̂*_j_*) for the encoded features ψ̌*_j_* = 0 and θ̌*_j_* = 0. We further amended this histogram by flipping at ψ̂*_j_* = 0 and θ̂*_j_* = 0 along the cue and report dimension, respectively, and adding all flipped versions. This makes use of our knowledge that the underlying distribution is symmetrical, and ensures symmetry in the estimate.

We then extended the histogram into a probability distribution *p̂*(ψ̂*_j_*, θ̂*_j_*) over the continuous, two-dimensional space of decoded cue and report values by bilinear interpolation and normalization of the result. This yields an estimate of the true distribution of decoding probabilities *p*(ψ̂*_j_*, θ̂*_j_*). Finally, we can determine the decoding probability for any given pair of encoded values by using the symmetry properties of the neural population model as follows (Eq. 19):

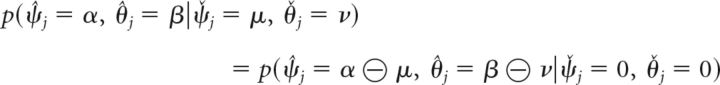
 Here and in the following, ⊕ and ⊖ denote addition and subtraction in circular space, respectively, yielding results in the range [−π, π).

##### Computing response probabilities.

To fit the model to the experimental data, we need to compute the probability *p*(θ̂*_u_* = θ) that the model will generate the response value θ for a given set of memory items and a given cue value (note that we omit the dependence on encoded feature values and cue value for brevity in the following equations). By marginalizing over the memory item *u* selected for response generation (Eq. 5), we can describe this probability as follows (Eq. 20):

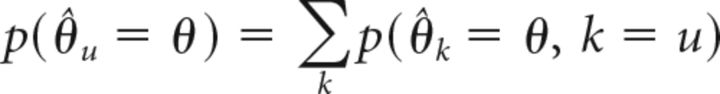
 The probability that item *k* is selected for response generation depends only on the decoded cue value ψ̂*_k_* of that item, but the decoded values for cue and report dimension are generally not statistically independent in the population model. To separate the probabilities, we marginalize Equation 20 over the decoded cue value as follows (Eq. 21):

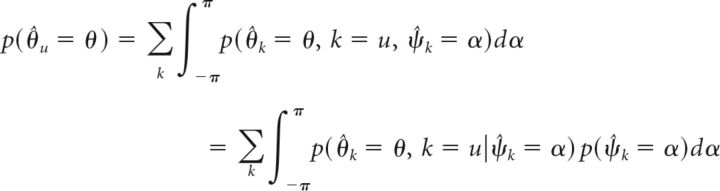
 For a given decoded value ψ̂*_k_* of item *k* in the cue dimension, the probabilities *p*(*k* = *u*) and *p*(θ̂*_k_* = θ) are conditionally independent, so we obtain the following (Eq. 22):

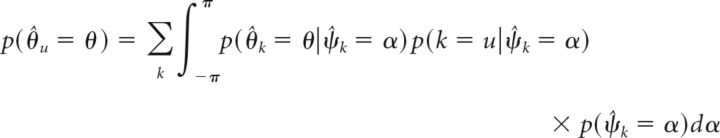
 The second term in the integral describes the probability that item *k* is selected for the response generation given the decoded cue dimension value ψ̂*_k_* for this item. Using Equation 5, we obtain for this term the following (Eq. 23):

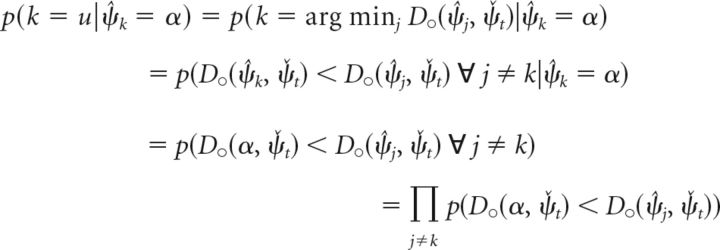
 We can determine the probability *p*(*D*_○_(α, ψ̌*_t_*) < *D*_○_(ψ̂*_j_*, ψ̌*_t_*)) by integrating the decoding probability over all values ψ̌*_j_* in circular space that satisfy the inequality, and obtain the following (Eq. 24):

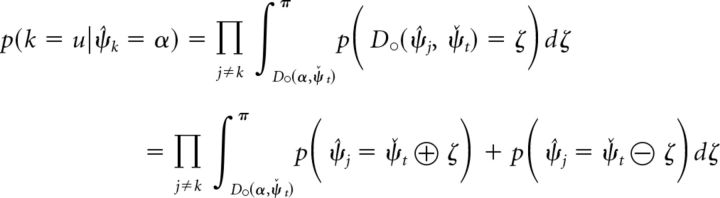
 We can derive the decoding probability for the cue dimension and the conditional decoding probability for the report dimension (used in Eq. 22) from the joint decoding probability as the following (Eqs. 25 and 26):

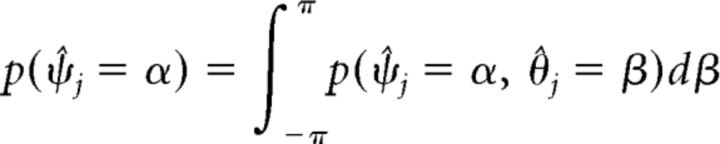


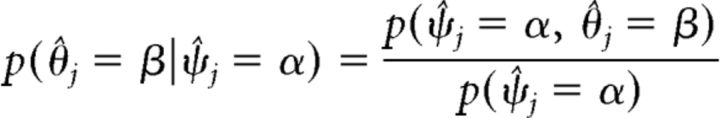
 With this, we can numerically evaluate Eq. 22 to compute the response probability *p*(θ̂*_u_* = θ) using the estimated decoding probabilities *p̂*(ψ̂*_j_*, θ̂*_j_*) obtained by sampling.

In addition, we can use the model to estimate which item from the sample array is selected for the response generation given the actual response θ̂*_u_* based on the individual summands in Eq. 20. This is expressed as follows (Eq. 27):

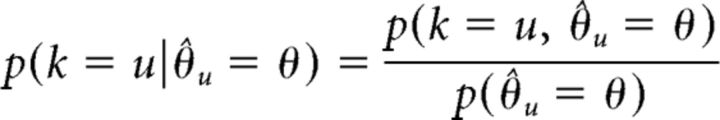


##### Fitting experimental results.

The population model for binding two feature dimensions has three parameters: the population gain, γ, and the cue-dimension and report-dimension tuning widths, κ_ψ_ and κ_θ_. We defined a 16 × 16 × 16 grid of parameter values (with γ exponentially spaced in the range [2^5.25^,2^9.25^], and κ_ψ_ and κ_θ_ each exponentially spaced in the range [2^−3^,2^2.25^]). For each triple of parameter values on this grid, we obtained an estimate of the population decoding probabilities *p*(ψ̂*_j_*, θ̂*_j_*) by sampling 10^6^ pairs of decoded values as described above.

For the color-location binding tasks (Exp. 1), we considered two versions of the model. The joint model assumes that a single neural population is used to generate responses in both task conditions. The model consequently has three free parameters, γ, κ_color_, and κ_location_; the width parameters were substituted appropriately for κ_ψ_ and κ_θ_ on report-color and report-location trials. The independent model fits the two experimental conditions using two separate population representations with different sets of parameters, yielding six parameters in total: γ^color^, κ_ψ_^color^, κ_θ_^color^, γ^location^, κ_ψ_^location^, κ_θ_^location^.

We also considered two models for the double-report task (Exp. 2). Both models are derived from the joint model for color-location binding, and they each have four parameters, γ, κ_color_, κ_location_, and κ_orientation_. In this task, subjects must report both the location φ_location_ and one nonspatial feature φ_feature_ of the target item (color or orientation), given the target's remaining nonspatial feature φ_cue_ as a cue. In the direct-binding model (Fig. 3*A*), the two report values are assumed to be generated independently from the cue, using one population code to associate cue feature to location, and a second one to associate cue feature to report feature. This yields three populations to cover both task conditions, representing color–location, orientation–location, and color–orientation associations. We can describe the response probability in this case as follows (Eq. 28): *p*(φ_location_, φ_feature_|φ_cue_) = *p*(φ_location_|φ_cue_)*p*(φ_feature_|φ_cue_), where both terms on the right-hand side are computed as in Eq. 22 for populations with feature dimensions substituted appropriately. In the spatial-binding model (Fig. 3*B*), it is assumed that two populations exist that each bind item location to one nonspatial feature. In the double-report task, the model first estimates the target item's location using the cue feature, and then uses the estimated location as a cue to retrieve the target's nonspatial report feature. We can write the response probability for this case as follows (Eq. 29): *p*(φ_location_, φ_feature_|φ_cue_) = *p*(φ_location_|φ_cue_)*p*(φ_feature_|φ_location_).

In both models, we assume that tuning curve widths for the same feature dimension are equal across different populations.

In addition, we fit a reduced model only to the spatial responses from Experiment 2 to detect spatial swap errors. The model uses two populations, each associating the cue feature (color or orientation) in the respective task condition to the item's location. The model does not make any assumptions about the binding between color and orientation, and is compatible with both the spatial-binding and the direct-binding model (which do not differ in how the spatial response is generated from the cue).

We determined a maximum likelihood fit of each model considered for the respective experiment to the behavioral data of each individual subject. We did this by computing the probability of obtaining the subject's actual response from the model in each trial for all different sets of parameter values. Response probabilities were computed numerically from the estimated decoding probabilities, using 180 sampling points along each feature dimension. Models were compared using the Akaike Information Criterion, with a correction for finite sample size (AICc).

For plotting the model results, we simulated 10^6^ trials with the best-fitting model parameters for each subject. We generated random target and nontarget feature values in the same way as in the behavioral study, and determined the response under the model by drawing from the estimated distributions of decoding probabilities. We then averaged the resulting histogram values over all subjects.

## Results

### Experiment 1: color-location binding

To investigate the nature of binding between spatial and nonspatial features, we presented participants with stimulus arrays consisting of randomly colored items at random locations on a circle (Fig. 1*A*). Participants were tested on their ability to recall the location of an item when cued by its color (report-location condition), or the color of an item when cued by its location (report-color condition). We use the results to test whether a memory representation with a single conjunctive population code can account for recall performance in both feature dimensions.

**Figure 4.**
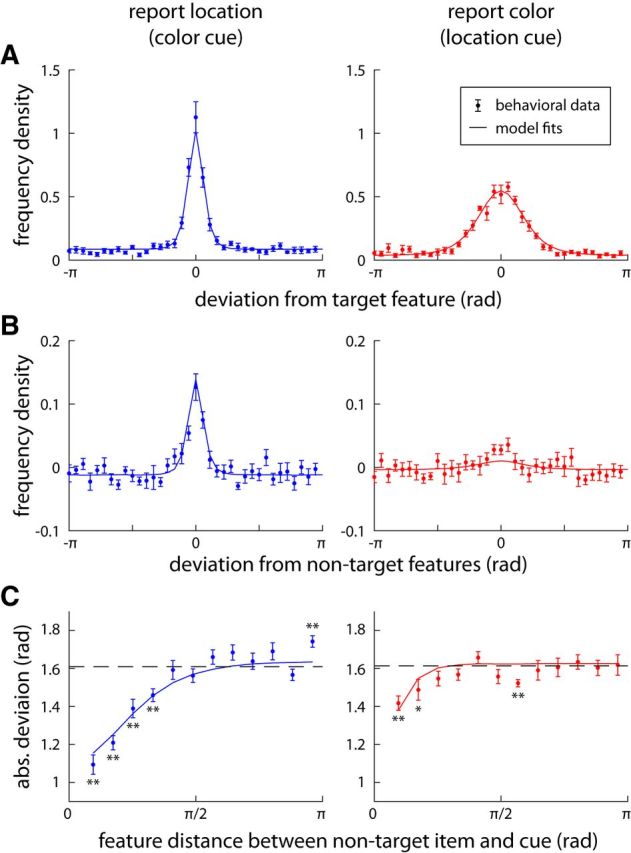
Behavioral results and model fits for Experiment 1. ***A***, Distribution of errors in reporting location (left) or color (right) of a cued item from the sample array. For all plots, data points show behavioral results (error bars indicate ±1 SE), solid curves show mean results from the fitted population coding model (joint model). ***B***, Deviation of responses from nontarget (i.e., uncued item) feature values. Histograms are corrected by subtracting distribution of deviations expected by chance (assuming no influence of nontargets on response; see Materials and Methods). Central tendency indicates the presence of swap errors, in which a nontarget feature is reported in place of the target. ***C***, Absolute deviation of responses from nontarget value as a function of similarity between nontarget and target in cue dimension (i.e., color in left plot, location in right plot). Dashed line indicates chance level. Asterisks indicate significant deviations from chance (*p < 0.05; **p < 0.01). Note that swap-error probability falls to chance as target and no-target cue-dimension features become more dissimilar.

Data points in Figure 4*A* plot the distribution of errors in reporting location (left) and color (right). Despite substantial differences in shape, the two distributions did not differ significantly in variability as measured by SD (location: σ = 1.06; color: σ = 1.16; *t*_(7)_ = 1.5, *p* = 0.17).

Figure 4*B* (data points) plots the distribution of angular deviation of responses from the other, nontarget items in each display (with a correction for chance; see Materials and Methods). If all responses were noisy estimates of the target, these distributions would be uniform; central tendency in these distributions indicates the presence of swap errors, in which a nontarget is reported in place of the target. The nontarget distribution in the report-location condition (left) displayed a strong central tendency (mean absolute deviation 1.52 vs chance 1.61, *t*_(7)_ = 7.2, *p* = 0.0002). The report-color distribution (right) had a statistically significant, but weaker, central tendency (mean absolute deviation 1.57 vs chance 1.61, *t*_(7)_ = 2.4, *p* = 0.047).

The differences in central tendency between nontarget distributions suggest that swap errors were common in recalling locations, but less so in recalling colors. Because swap errors appear uniformly distributed relative to the target, the error distribution in Figure 4*A*, left, is consistent with a high-precision representation of target location (the sharp central distribution) combined with a high frequency of swap errors (the long tails), whereas the broad distribution in Figure 4*A*, right, is consistent with a lower-precision representation of target color, with fewer swap errors.

Data points in Figure 4*C*, left, show how the deviation of location estimates from a nontarget's location varies with the similarity of that nontarget's color to the color of the target. If there were no swap errors, these data would follow the dashed line, indicating the deviation expected by chance; values below the line indicate that responses are biased toward the nontarget value, which is consistent with the presence of swap errors. In fact, the absolute deviation was significantly lower than chance (asterisks indicate significance) but only when target and nontarget had similar colors. Correspondingly, the right plot in Figure 4*C* shows how the deviation of color estimates from a nontarget's color varies with the similarity of that nontarget's location to the location of the target. This analysis reveals the presence of swap errors in the report-color condition only for nontargets that are very similar to the target in the cue feature, i.e., are very close together in space.

### Model fits

We fit the behavioral data with a neural population model that uses conjunctive coding to capture the binding between color and location for each stimulus (Fig. 2). The neural population is characterized by three free parameters, namely the widths of the von Mises tuning curves for the two feature dimensions, and the gain factor. We fit two variants of the model to the behavioral data: in the joint model, a single neural population is used to capture both conditions of the task by changing only the feature dimension (color or space) that takes the role of cue feature and the feature dimension that takes the role of report feature; in the independent model, two separate populations are fit to the report-color and report-location conditions, yielding a total of six free parameters. Models are fit to the data by maximum likelihood estimation, determining the parameters that maximize the model's response probability (Eq. 22) for the subjects' actual response values over all trials.

We compared the model fits by their AICc values. The joint model achieved slightly lower AICc values (mean ΔAICc = 0.69), indicating a better fit after adjustment for the number of free parameters, although this result was not statistically significant across subjects (*t*_(7)_ = 0.42, *p* = 0.69; *t* test performed after finding no significant deviations from normality in AICc scores for each model using Lilliefors test). We simulated the experiment with the best-fitting joint model for each subject to generate distributions of response errors, and obtained close quantitative fits (Fig. 4*A*, solid lines). The different error distributions for the two conditions in the model can be attributed to the widths of tuning curves for color and location. The model fits show significantly sharper tuning curves for location (mean concentration parameter across subjects κ = 2.95) than for color (κ = 0.44, *t*_(7)_ = 4.39, *p* = 0.003). The sharper tuning curves produce on average smaller errors in decoding the memorized feature values (given otherwise equal parameters and assuming uniform coverage of the feature space), accounting for the sharper central peak in the error distribution for the report-location condition.

The model also reproduces the central tendency in response deviations from nontarget items (Fig. 4*B*, solid line) that indicates the occurrence of swap errors. In the model, a swap error occurs if a nontarget item is estimated to be more similar to the given cue than the target (which actually matches the cue exactly). This can happen due to decoding errors in the cue-feature dimension, and is particularly likely for nontarget items that are similar to the target in their cue feature (since for these even a relatively small decoding error can lead to a swap error). This mechanism accounts for the empirically observed effect of cue similarity on swap errors, which is quantitatively reproduced by the model (Fig. 4*C*, solid line).

The proportion of swap errors is directly influenced by the width of the tuning curves for the cue feature, since a wider tuning curve produces a larger mean decoding error. In the model simulations, we can directly measure the proportion of swap errors. For the report-color condition (cued by location with sharper tuning curves), the best fitting model produced swap errors in 15.8% of trials, versus 50.5% of trials in the report-location condition (cued by color with wider tuning curves; significantly different, *t*_(7)_ = 12.1, *p* < 0.001).

### Experiment 2: binding between nonspatial features

The results of the first experiment indicate that a conjunctive coding model can quantitatively reproduce empirical patterns of errors in binding a spatial and nonspatial feature. The aim of the second experiment was to test competing models of binding between two nonspatial features. We presented participants with arrays of randomly colored, randomly oriented bars at random locations on a circle (Fig. 1*B*). In the orientation-cue condition, participants were given the orientation of one item from the sample array as a cue and had to sequentially report that item's color and its location. In the color-cue condition, a color was given as cue and participants had to report the orientation and the location of the matching item. The order of the reports in both conditions was balanced across blocks. We pooled results over report orders (location first or nonspatial feature first) in each condition after finding no significant effect of report order on the SD of responses (multivariate ANOVA, orientation-cue: Wilk's λ = 0.861, *F*_(2,13)_ ≈ 1.05, *p* = 0.38; color-cue: Wilk's λ = 0.79, *F*_(2,13)_ ≈ 1.75, *p* = 0.21).

**Figure 5.**
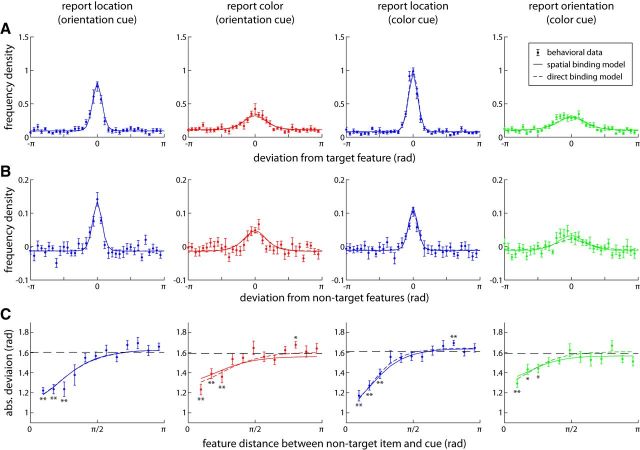
Behavioral results and model fits for Experiment 2. Location (blue) and color (red) responses from the orientation-cue condition are shown in the first and second column, respectively. Location and orientation (green) responses from the color-cue condition are shown in the third and fourth column, respectively. Orientation values are scaled up to the range [−π, π) to allow easier comparison between conditions. Solid lines show model fits with the spatial-binding model; darker dashed lines show fits with the direct-binding model. ***A***, Distribution of report error (deviation from target feature value). ***B***, Deviation of responses from nontarget (i.e., uncued item) feature values, corrected by subtracting distribution expected by chance. ***C***, Mean absolute deviation of responses from nontarget values as a function of similarity between the nontargets and the target in the cue dimension. Dashed line indicates chance level.

Figure 5*A* plots the distribution of errors in reporting location and color in the orientation-cue condition (first and second column), and the distribution of errors when reporting location and orientation in the color-cue condition (third and fourth column). In both conditions, the error distribution for location showed a significantly lower SD than the error distribution for the nonspatial feature (orientation-cue: σ = 1.40 vs σ = 1.70, *t*_(7)_ = 4.5, *p* = 0.003; color-cue: σ = 1.16 vs σ = 1.52, *t*_(7)_ = 7.2, *p* < 0.001). The SD for reporting location was significantly lower when cued with color than when cued with orientation (*t*_(7)_ = 2.5, *p* = 0.040), indicating that the color cue could be used more effectively for reporting the target location. We further note that the SD for location in the color-cue condition closely matched the SD in the report-location condition from Experiment 1 (these conditions are the only ones that are directly analogous between the two experiments; unpaired *t* test: *t*_(14)_ = 0.08, *p* = 0.94). This indicates that the additional task of memorizing and reporting orientations did not significantly interfere with the spatial-recall task.

To test for swap errors, we determined the response deviations from all nontarget items, shown in Figure 5*B* (with correction for chance). A pronounced central peak is visible in the distributions for all report conditions, and comparisons of mean absolute deviations to values expected by chance confirmed a significant central tendency for all reports (orientation-cue, location: *t*_(7)_ = 6.8, *p* < 0.001; color: *t*_(7)_ = 4.3, *p* = 0.003; color-cue, location: *t*_(7)_ = 10.6, *p* < 0.001; orientation: *t*_(7)_ = 4.7, *p* = 0.002). This strongly indicates the occurrence of swap errors in all reports.

Figure 5*C* shows the effect of cue similarity on swap errors, plotting the mean absolute deviation of the report value from a nontarget's feature against the difference in cue feature value between that nontarget and the target. In all report conditions, we found evidence for swap errors for nontargets that were similar to the target in their cue feature (mean absolute deviation significantly different from chance value, shown as dashed line in Fig. 5*C*), but not for those that were dissimilar. The range of cue feature values for which swap errors occurred was comparable for reporting the spatial and the nonspatial feature in each condition. This is consistent with the hypothesis that swap errors are driven by similarity in the cue feature, without influence of the report feature.

### Model fits

We considered two possible models for the binding of multiple feature dimensions tested in this experiment. The direct-binding model (Fig. 3*A*) employs neural populations that represent all possible combinations of two features, and uses two of these in each task condition to independently generate the spatial and nonspatial responses. The spatial-binding model (Fig. 3*B*) only employs populations for color-location and orientation-location binding. The model generates the spatial response directly from the given cue, and then uses the estimated item location as a cue to generate the nonspatial response. Both models have four free parameters, namely the tuning curve widths for the three feature dimensions, and a global gain factor.

We obtained maximum likelihood fits of the behavioral data for both models and computed their AICc scores. The spatial-binding model reached significantly higher likelihood values and consequently lower AICc scores across subjects, indicating that it provides a better explanation for the observed results (mean ΔAICc = 98.8; *t*_(7)_ = 6.2, *p* < 0.001; Lilliefors test found no significant deviation from normality in AICc scores). Simulation results obtained with the best-fitting model provide close fits of the experimentally observed distributions of response errors, response deviations from nontarget items, and effect of cue similarity on swap errors (Fig. 5, solid lines). We note that in the spatial-binding model, two types of swap errors can occur: the first type may happen when selecting the spatial response based on the cue feature, analogous to the swap errors observed in the report-location condition of Experiment 1. A second type can occur when the estimated spatial location is used to select the memorized item for the nonspatial response. Both types contribute to the resulting distributions of response errors in the model, although the second type is less frequent due to significantly sharper tuning curves for location than for nonspatial features (27% of trials in both conditions vs 55% for orientation-cue and 49% for color-cue). The higher proportion of swap errors when cued with orientation is consistent with the finding that orientation was a less effective cue for the spatial response in the experimental results.

In the best-fitting model, the mean concentration parameter of neural tuning curves for the spatial dimension (κ = 2.22) was significantly higher than concentration parameters for both color (κ = 0.45, *t*_(7)_ = 4.73, *p* = 0.002) and orientation (κ = 0.36, *t*_(7)_ = 4.71, *p* = 0.002). We also found a significant difference between concentration parameters for color and orientation (*t*_(7)_ = 2.91, *p* = 0.023), accounting for the higher proportion of swap errors when cueing with orientation than with color. We note that these values cannot be compared directly to the corresponding values from Experiment 1 since the model fits also differ in their gain parameters.

### Error correlations

While the comparison of AICc scores clearly favors the spatial-binding model, we observed that model simulations based on the best-fitting direct-binding model produced nearly identical fits to the error distributions and other plots shown in Figure 5 (darker dashed lines). This raises the question: what causes the difference in AICc scores for the two models? One key difference between the two models is the pattern of swap errors across the two responses they predict. In the direct-binding model, if a swap error occurs in the generation of the spatial response, this has no effect on the response for the nonspatial feature (Fig. 3*A*). A swap error may occur here as well, but it would be independent of the swap error for the spatial response. In contrast, in the spatial-binding model, a swap error in the spatial response means that the location of the selected nontarget item will be used for the generation of the nonspatial response (Fig. 3*B*). The nonspatial response should then be centered on the feature value of the nontarget at the selected location, rather than the target. In particular, this mechanism predicts a strong correlation between swap errors, and consequently absolute response errors, in spatial and nonspatial responses.

To test this, we determined Pearson's product–moment correlation coefficient for absolute response errors in the spatial and nonspatial response across trials for all subjects. Correlation coefficients were significantly >0 in both the orientation-cue (mean across subjects: *r* = 0.31; *t*_(7)_ = 6.77, *p* < 0.001) and the color-cue condition (*r* = 0.33, *t*_(7)_ = 9.66, *p* < 0.001). These values closely match the predictions of the spatial-binding model (orientation-cue: *r* = 0.34, not significantly different, *t*_(7)_ = 0.67, *p* = 0.52; color-cue: *r* = 0.30, not significantly different, *t*_(7)_ = 0.82, *p* = 0.44). The direct-binding model predicts significantly lower values for both task conditions that do not match the experimental findings (orientation-cue: *r* = 0.016, *t*_(7)_ = 6.63, *p* < 0.001; color-cue: *r* = 0.025, *t*_(7)_ = 8.98, *p* < 0.001).

**Figure 6.**
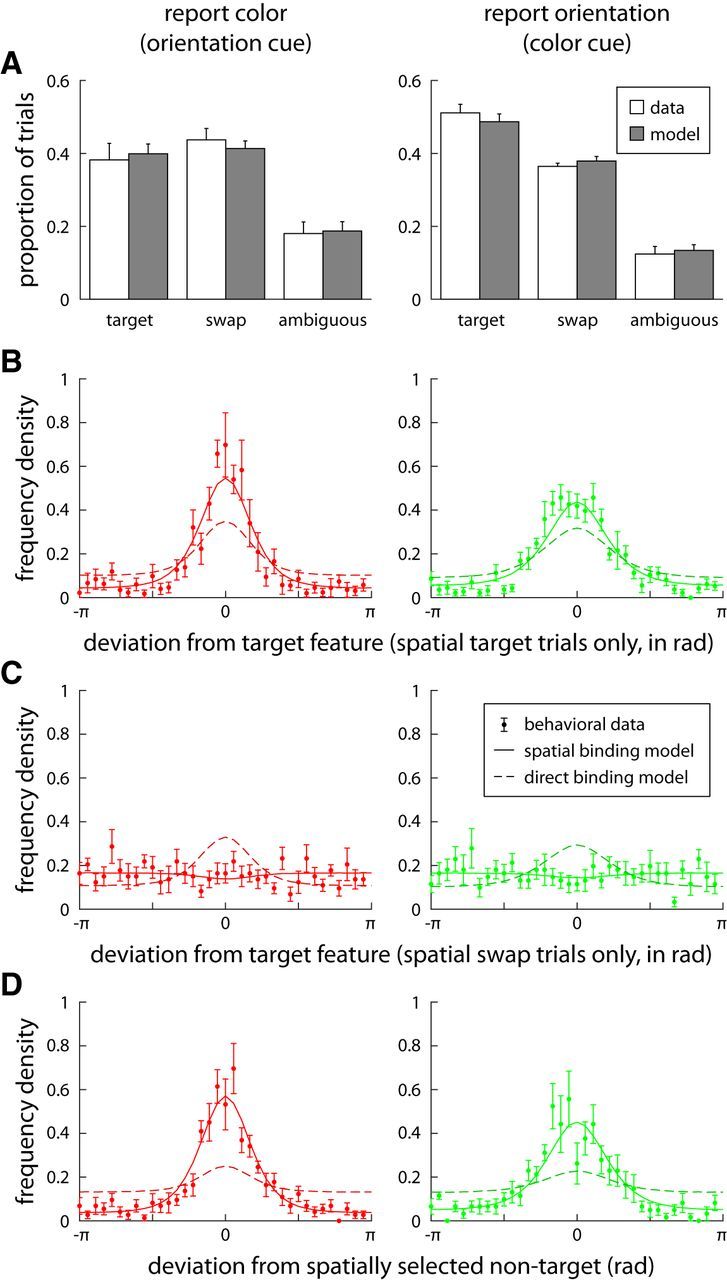
Results for nonspatial responses in Experiment 2 grouped by occurrence of spatial-swap errors. Results from the color report in the orientation-cue condition are shown in the left column. Results from the orientation report in the color-cue condition are shown in the right column. ***A***, Proportion of trials in which the spatial response was classified as response to target, swap error, and ambiguous. White bars show results for behavioral data and gray bars show results for model simulations of the spatial-binding model. ***B***, Distribution of response errors for the nonspatial feature in trials classified as spatial target trials. Solid lines in all plots show mean results from fitted spatial-binding model. Darker dashed lines show mean results from direct-binding model. ***C***, Distribution of nonspatial response errors for trials classified as spatial swap trials. ***D***, Deviation of nonspatial responses from the feature value of the spatially selected item in spatial swap trials.

We used the population model to directly investigate response error distributions for the nonspatial feature in spatial swap trials (i.e., trials in which a swap error occurred in the generation of the spatial response) and spatial target trials (in which the spatial response was directed to the target). To this end, we fit a reduced population model (equivalent to the model for Exp. 1) only to the spatial responses of each subject and used this model to identify spatial swap and spatial target trials in the experimental results (see Materials and Methods). We applied the same analysis to the spatial responses generated by the population models to allow a direct comparison between experimental and model results. A majority of trials in both conditions can be classified as either spatial swap or spatial target trials, and the estimated proportions for the model simulations closely match those for the experimental results (Fig. 6*A*).

We first analyzed error distributions for the nonspatial response in trials classified as spatial target trials (Fig. 6*B*). Error distributions were narrower in spatial target trials than over all trials of the same condition, with a significantly lower SD (orientation-cue: σ = 0.99 vs. σ = 1.70, *t*_(7)_ = 8.93, *p* < 0.001; color-cue: σ = 1.03 vs. σ = 1.52, *t*_(7)_ = 8.61, *p* < 0.001). This is consistent with the prediction of the spatial-binding model, which provides a close fit to these error distributions (Fig. 6*B*, solid lines). In this model, the location from the spatial response is used to generate the nonspatial response. Thus, if this spatial response is directed at the correct item, then swap errors based on similarity in the original cue (which make up most swap errors) are excluded in the nonspatial response. The direct-binding model does not predict such an effect, and fails to reproduce the narrower error distributions for spatial target trials (Fig. 6*B*, dashed lines).

Next, we analyzed error distributions in the nonspatial response for spatial swap trials, shown in Figure 6*C*. In both conditions, the error distribution is largely flat, with no apparent central peak. By comparing the mean absolute response error to the error expected for uniformly distributed random responses, we confirmed that there is no significant central tendency in the response distributions for spatial swap trials (orientation-cue: *t*_(7)_ = 0.31, *p* = 0.76; color-cue: *t*_(7)_ = 1.29, *p* = 0.24). The spatial-binding model reproduces this flat distribution (Fig. 6*C*, solid lines). Here, a spatial swap error means that the location of a nontarget item will be used to generate the nonspatial response, which can consequently only by chance match the target feature. The experimental results are inconsistent with the direct-binding model, which predicts that the nonspatial response should be unaffected by spatial swap errors (dashed lines).

For the spatial swap trials, we additionally analyzed the deviations of the nonspatial response from the feature value of the nontarget item selected for the spatial response. The resulting distributions (Fig. 6*D*) show a pronounced central peak, and have SDs lower than the error distributions over all trials (orientation-cue: σ = 1.01, *t*_(7)_ = 7.47, *p* < 0.001; color-cue: σ = 1.07, *t*_(7)_ = 7.53, *p* < 0.001). They are well fit by the spatial-binding model (solid lines), which predicts that these distributions should be equal to the error distributions in spatial target trials (Fig. 6*B*). Since the nonspatial response in this model is based on the location of the spatial response, it should be centered on the feature value of the spatially selected item, independent of whether that item is the target or a nontarget item. Indeed, we found no significant difference in the experimental results between the error distribution for spatial target trials (Fig. 6*B*) and the distribution of deviations from the spatially selected item in spatial swap trials (Fig. 6*D*; orientation-cue: *t*_(7)_ = 0.31, *p* = 0.77; color-cue: *t*_(7)_ = 0.68, *p* = 0.52). The results are again inconsistent with the direct-binding model (Fig. 6*D*, dashed lines), which predicts only a weak central peak reflecting the small proportion of trials in which, by chance, the same swap error occurs independently in the spatial and nonspatial response.

We note that these results are reproduced when analyzing only trials in which the nonspatial response is produced first, and the spatial response second. They can therefore not be attributed to the spatial response forcing the selection of a memorized item before the nonspatial response is initiated.

## Discussion

It has long been recognized that memorizing the binding between visual features is an additional challenge over and above memorizing the features themselves (Treisman, 1996; Wheeler and Treisman, 2002). In change-detection tasks, this challenge is reflected in specific failures to detect changes that only affect feature conjunctions (Treisman and Zhang, 2006), while in cued recall tasks it is reflected in swap errors (Bays et al., 2009; Bays, 2016).

We presented a model of feature binding that combines neural population representations with conjunctive coding. The population model is related to approaches linking working-memory performance to sustained neural activity (Wei et al., 2012; Johnson et al., 2014), but focuses on simplicity over biophysical detail and employs only a static representation of population activity. It has previously been shown that maximum likelihood decoding from such a population code for a single visual feature, with stochasticity induced by random noise in the neural activity, can account for precise patterns of error distributions in cued recall tasks (Bays, 2014). This model successfully accounted for decreasing recall precision with increasing set size through normalization of total spiking activity in a population representing all memorized items.

In the extended population model with conjunctive coding for cue and report features, swap errors can be explained by decoding errors in the cue dimension, directly analogous to decoding errors in the report dimension. Such decoding errors cause a nontarget item to be judged as the one most similar to the cue, and the associated report feature to be produced as a response. The model thereby provides an integrated account for different types of errors in cued recall tasks, based on noise in neural populations. The different patterns of response errors in the two conditions of Experiment 1—sharp distributions around the target location combined with a large proportion of swap errors in the case of spatial responses, wider distributions with fewer swap errors for color responses—can be fully explained in this model by different widths of neural tuning curves for the two feature dimensions.

A theoretical investigation of feature binding using a similar conjunctive population code has previously been presented by Matthey et al. (2015), with several differences in the implementation. The earlier model employed a mixed code that includes neurons selective for a single-feature dimension, which may contribute to the efficient memorization of individual features. It also explicitly combined the representations of all items into a single population representation (a “palimpsest” model), which we expect to reflect the representations in the biological system more closely. We omitted these aspects to make the analysis of swap errors in individual trials mathematically feasible, whereas the previous work only analyzed expected rates of different errors. The model of Matthey et al. also proposed a conjunctive code for color–orientation associations, in contrast to the spatial-binding model favored by the present results. We note, however, that the differences between these models only become apparent in the correlations between spatial and nonspatial responses, which were not addressed in the previous work.

The model of Matthey et al. made the qualitative prediction that swap errors should occur specifically for items that are similar to the target with respect to the cue feature. Such an effect has previously been described for spatial proximity when the target is cued by locations (Emrich and Ferber, 2012; Rerko et al., 2014; for a meta-analysis, see Bays, 2016). Here, we have experimentally confirmed the cue similarity effect independent of the feature dimension used for the cue, and quantitatively accounted for the effects in the population model.

Using a cued recall task with both spatial and nonspatial report features, we found strong evidence for a spatial-binding mechanism, in which color and orientation of each object are separately bound to location, and are linked only via their shared location. For trials with swap errors in the spatial response, the response in the nonspatial feature was strongly centered on the feature value of the spatially selected item, with no indication that the nonspatial report feature could be retrieved in any way other than via its location. The most parsimonious explanation for these experimental results is that nonspatial features are bound only via space. This interpretation is consistent with analogous findings at the perceptual level (Nissen, 1985), and supported by the observation that spatial attention is engaged when retrieving items from working memory even when cued by nonspatial features (Theeuwes et al., 2011).

These results are particularly informative if we contrast them with experiments in which a spatial cue is used to retrieve multiple nonspatial features of an object (such as color and orientation). Using tasks and analyses comparable to the ones employed here, previous studies have consistently found only weak correlations between different nonspatial reports (Bays et al., 2011a; Fougnie and Alvarez, 2011; Fougnie et al., 2013). This is consistent with the present model in which space mediates the binding of nonspatial features. When cued with location, the nonspatial features are independently retrieved from the separate feature maps; but when cued with orientation, the color of the cued item can only be retrieved via the item's location.

The earlier findings of low error correlations when using a spatial cue rule out several alternative accounts of binding in visual working memory. This includes accounts of working memory that assume the coherent memorization of only a subset of objects in a bound representation (Luck and Vogel, 1997); models like the binding pool (Swan and Wyble, 2014), in which location and nonspatial features are equally bound to object tokens; and models based on binding through synchrony of neural spiking activity (Raffone and Wolters, 2001). In all these models, it should be possible to retrieve a coherent memory representation of a single object given one of its features as a cue, predicting high correlations in swap errors independent of whether the cue is spatial or nonspatial. While such a mechanism is compatible with the present findings of high error correlations when using nonspatial cues, it contradicts the earlier results of low error correlations when using a spatial cue. In combination, these results strongly support a special role for space in feature binding.

Binding via space as a general principle is plausible with respect to the available neural data, which show that neurons responsive to visual features, such as color or shape, almost universally retain a sensitivity to stimulus location as well (Op De Beeck and Vogels, 2000). This type of binding also avoids the combinatorial explosion of required representational resources that would result if every possible feature combination were represented through conjunctive coding.

A special role of space in feature binding has already been suggested in the influential Object File Theory (Kahneman et al., 1992), but without specifying a concrete mechanism. The present approach using separate, spatially bound feature maps for color and orientation is consistent with the idea that separate memory stores with largely independent capacity limits exist for different features (Wheeler and Treisman, 2002; Bays et al., 2011; Wang et al., 2017), without requiring an inherently bound representation based on objects rather than features (Luck and Vogel, 1997). This idea is supported by recent findings showing no object-specific benefit in working memory, but rather a benefit based on the number of individual locations at which visual features appear (Wang et al., 2016).

The concept of separate feature maps linked via space is well established at the perceptual level, such as in the Feature Integration Theory (Treisman, 1988) and in neural models of visual search (Itti and Koch, 2001; Hamker, 2005; Wolfe, 2007). Models based on dynamic neural fields have also proposed a similar architecture for visual working memory representations (Johnson et al., 2008; Schneegans et al., 2016). While these models describe a possible neural process for solving different types of change-detection tasks, they have not been used to actually fit behavioral data. However, the latter model raises the relevant question of which spatial-reference frame is used for feature maps in working memory—retinotopic (which would require a form of remapping to compensate for gaze changes) or gaze invariant (requiring a spatial transformation of the visual scene). The present work does not address this question since gaze direction was fixed in the modeled task.

Another critical question is how a spatial-binding model can account for feature binding in tasks that use sequential presentation of stimuli at a single location (Gorgoraptis et al., 2011). This may require a reinterpretation of the spatial dimension in the model as a more general spatial–temporal representation that mediates feature binding. Alternatively, multiple items that occupy the same space may be internally remapped to unoccupied locations for memorization. It has been observed that sequential presentation of items at the same location specifically impairs memory for feature bindings (Pertzov and Husain, 2014), indicating that this situation does indeed pose a particular challenge for the neural system. Further research will be needed to fully characterize the spatial representations used in feature binding.
